# Coenzyme Q_10_ Protects Human Endothelial Cells from β-Amyloid Uptake and Oxidative Stress-Induced Injury

**DOI:** 10.1371/journal.pone.0109223

**Published:** 2014-10-01

**Authors:** Mario Durán-Prado, Javier Frontiñán, Raquel Santiago-Mora, Juan Ramón Peinado, Cristina Parrado-Fernández, María Victoria Gómez-Almagro, María Moreno, José Alberto López-Domínguez, José Manuel Villalba, Francisco J. Alcaín

**Affiliations:** 1 Department of Medical Sciences, Faculty of Medicine, University of Castilla-la Mancha, Ciudad Real, Spain; 2 Oxidative Stress and Neurodegeneration Group, Regional Centre for Biomedical Research, Ciudad Real, Spain; 3 Department of Organic Chemistry, Faculty of Chemical Sciences and Technologies and Instituto Regional de Investigación Científica Aplicada, Universidad de Castilla-La Mancha, Ciudad Real, Spain; 4 Fundación Parque Científico y Tecnológico de Albacete, Albacete, Spain; 5 Department of Cell Biology, Physiology and Immunology, Faculty of Sciences, University of Córdoba, Agrifood Campus of International Excellence ceiA3, Córdoba, Spain; IISER-TVM, India

## Abstract

Neuropathological symptoms of Alzheimer's disease appear in advances stages, once neuronal damage arises. Nevertheless, recent studies demonstrate that in early asymptomatic stages, ß-amyloid peptide damages the cerebral microvasculature through mechanisms that involve an increase in reactive oxygen species and calcium, which induces necrosis and apoptosis of endothelial cells, leading to cerebrovascular dysfunction. The goal of our work is to study the potential preventive effect of the lipophilic antioxidant coenzyme Q (CoQ) against ß-amyloid-induced damage on human endothelial cells. We analyzed the protective effect of CoQ against Aβ-induced injury in human umbilical vein endothelial cells (HUVECs) using fluorescence and confocal microscopy, biochemical techniques and RMN-based metabolomics. Our results show that CoQ pretreatment of HUVECs delayed Aβ incorporation into the plasma membrane and mitochondria. Moreover, CoQ reduced the influx of extracellular Ca^2+^, and Ca^2+^ release from mitochondria due to opening the mitochondrial transition pore after β-amyloid administration, in addition to decreasing O_2_^.−^ and H_2_O_2_ levels. Pretreatment with CoQ also prevented ß-amyloid-induced HUVECs necrosis and apoptosis, restored their ability to proliferate, migrate and form tube-like structures *in vitro*, which is mirrored by a restoration of the cell metabolic profile to control levels. CoQ protected endothelial cells from Aβ-induced injury at physiological concentrations in human plasma after oral CoQ supplementation and thus could be a promising molecule to protect endothelial cells against amyloid angiopathy.

## Introduction

Alzheimer disease (AD) is a chronic neurodegenerative pathology characterized by the proteolytic processing of the amyloid precursor protein to form amyloid peptide (Aβ), which aggregates into extracellular amyloid plaques to cause neurotoxicity and the progressive cognitive decline typical of the disease in a process known as the “amyloid cascade” [1]. However, evidence indicates that circulating soluble Aβ exerts biological effects in AD patients prior to neuronal injury, much earlier than the onset of cognitive deficits. Indeed, soluble circulating Aβ damages endothelial cells in asymptomatic Alzheimer's stages preceding Aβ deposition [2]. In this sense, circulating Aβ affects the blood brain barrier to compromise its permeability and integrity [3] while also impairing blood supply by damaging small blood vessels, weakening oxygen exchange and nutrient delivery [4].

The concept of endothelial injury preceding neuronal damage is reinforced, first, by the fact that endothelial cells are the first line of contact with circulating Aβ, second, by the observation that oxidative stress in cerebral blood vessels occurs when there is no evidence of Aβ deposition in cerebral parenchyma and blood vessels, and third, by the observation that endothelial cells are more sensitive to Aβ and its active fragment Aβ_25–35_ than neurons or smooth muscle cells [2], [5]–[7]. Indeed, both Aβ and Aβ_25–35_, exert a prominent pro-apoptotic and necrotic effect on endothelial cells by mechanisms that involve an increase in free cytosolic calcium concentration ([Ca^2+^]_i_) and reactive oxygen species such as O_2_^.−^ and H_2_O_2_ [5], [8]–[11]. Although the exact mechanism of Aβ-mediated [Ca^2+^]_i_ entry into endothelial cells is not completely understood, there is evidence in neurons pointing to the formation of pores that cause instability at the plasma membrane and produce ion leakiness, thus mediating a robust calcium influx into the cell [12]–[14]. Furthermore, Aβ increases the level of free oxygen radicals that react with nitric oxide (NO) and produces oxidative/nitrosative stress that alters vascular function [15]. Increased O_2_^.−^ and H_2_O_2_ produced by dismutation of these radicals also induces the opening of the permeability transition pore (mPTP), a mitochondrial membrane channel involved in cell death, thus damaging mitochondria and inducing necrosis and apoptosis [16]–[18]. This damage is further reinforced by trafficking and accumulation of the Aβ peptide into mitochondrial cristae [19].

Recently, a meta-analysis of randomized controlled trials in cardiovascular diseases, which are risk factors for AD in the elderly [20], provided evidence for effective treatment with the lipophilic antioxidant coenzyme Q_10_ (CoQ) to improve endothelial cell function [21]–[23]. Moreover, CoQ also prevented oxidative stress and calcium-mediated necrosis and apoptosis in human umbilical vein endothelial cells (HUVEC) exposed to damaging and pro-oxidizing stimuli such as high glucose concentration, oxidized LDL or angiotensin II [24]–[27]. Finally, it is known that a ubiquinone binding site regulates the aperture of the mPTP [28] and that CoQ is effective at inhibiting mPTP opening triggered by H_2_O_2_ in epithelial cells [29].

Based upon these observations, here we explored the role of CoQ against Aβ injury in endothelial cells using microscopy, biochemistry and metabolomic approaches. Our results show that pretreatment of endothelial cells with physiological concentrations of CoQ delays Aβ incorporation into the plasma membrane and its trafficking to mitochondria, reduces ROS production and calcium influx while increasing NO levels. Moreover, CoQ treatment impedes mPTP opening and reduces necrosis and apoptosis, while also restoring cell migration, wound healing and cell tube formation to control conditions. CoQ blocks Aβ-induced changes in the endothelial metabolic profile. In sum, CoQ could be a promising molecule to treat endothelial dysfunction associated with early, asymptomatic AD stages in high-risk populations.

## Results

### CoQ prevents β-amyloid-induced endothelial cell death and restores migration and angiogenesis *in vitro*

**Figure 1:**
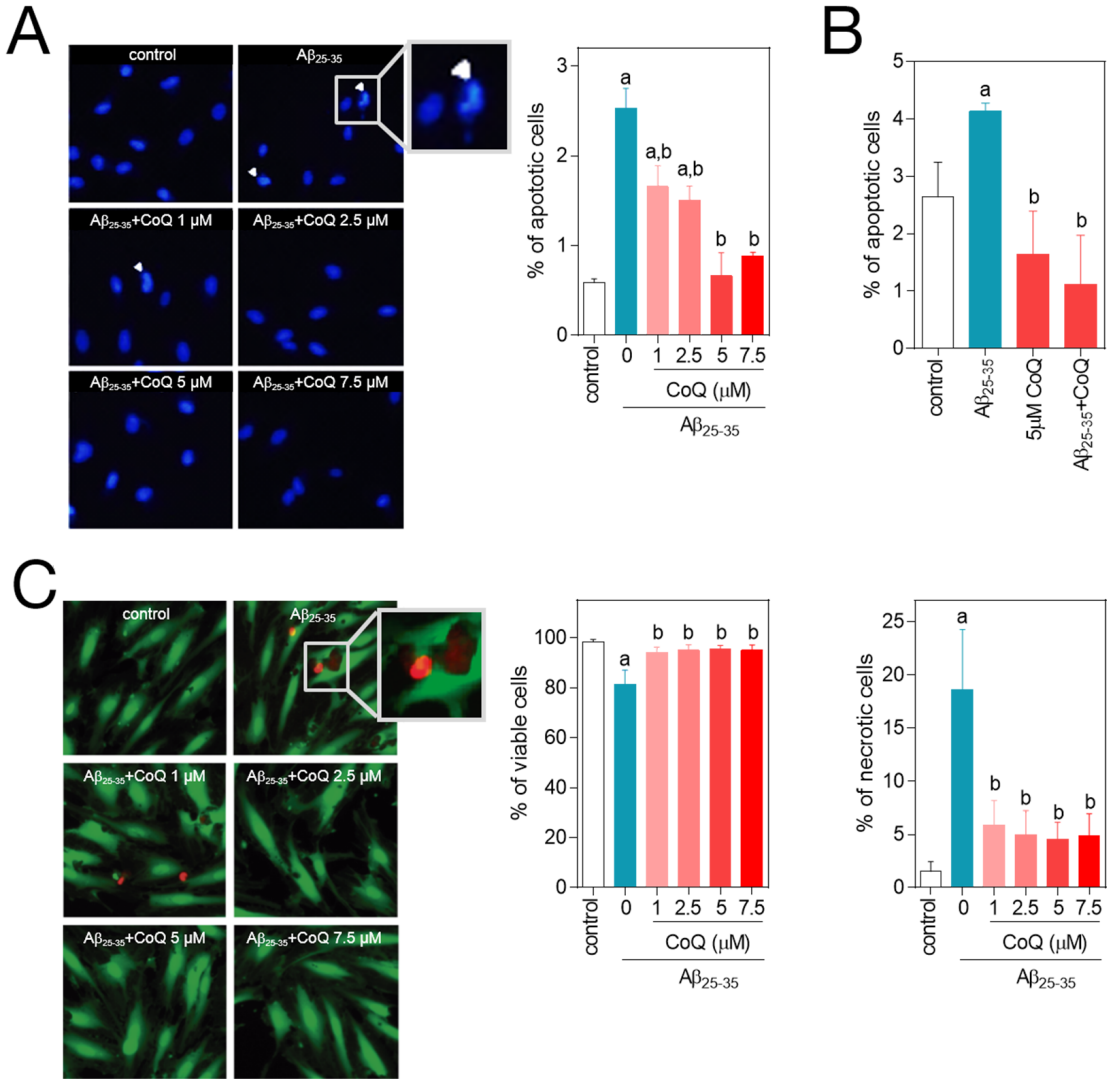
CoQ protects endothelial cells from β-amyloid-induced apoptosis and necrosis. HUVECs were incubated for 12 h with vehicle alone or with increasing CoQ concentrations (1 to 7.5 µM) and then treated for additional 24 h with 5 µM Aβ_25–35_. **A**) Apoptosis was determined by DAPI staining and morphological analysis of nuclei. White arrows indicate typical apoptotic nuclei. Results are expressed as the percentage of apoptotic *vs*. total nuclei (300 cells/treatment, n = 3). **B**) Apoptosis was also evaluated by flow cytometry. Results are expressed as percentage of cells positive for Annexin V *vs.* total (n = 3). Viability and necrosis (**C, left and right, respectively**) were determined by cell co-staining with calcein-AM (green) and ethidium bromide (orange) and evaluated by qualitative fluorescence microscopy. Results are expressed as percentage of viable/necrotic cells *vs.* total (n = 3). a, *p*<0.05 vs. control; b, p<0.05 *vs*. Aβ_25–35_.

It was previously reported that cellular exposure to the amyloid fragment Aβ_25–35_ results in endothelial cell toxicity similar to β_1–42_, via inducing cell apoptosis and necrosis [5]. In our experiments we explored the effect of the Aβ_25–35_ fragment on cell death and the possible cytoprotective role of CoQ. The addition of 5 µM Aβ_25–35_ to HUVECs for 24 h increased the percentage of apoptotic nuclei from 0.5% to 2.6% (Figure 1A) and the percentage of Annexin V positive cells from 2.7% to 4.2%, (Figure 1B), indicating a modest but significant increase in apoptosis. A 12 h preincubation with CoQ resulted in a dose-dependent decrease of apoptotic nuclei, reaching control levels at 5 µM CoQ (Figure 1A), which was confirmed by flow cytometry determination of Annexin V (Figure 1B). The action of CoQ was cytoprotective, as cells needed to be pre-treated with the lipophilic antioxidant prior to Aβ_25–35_ addition for an observable effect. Simultaneous treatment with 5 µM Aβ_25–35_ and CoQ did not affect the percentage of apoptotic cells (Figure S1A). On the other hand, treatment with 5 µM Aβ_25–35_ produced an 18% decrease of HUVECs viability, which was mirrored by a similar increase in necrosis (Figure 1C). Preincubation with CoQ restored cell viability to control levels and prevented Aβ_25–35_ induced necrosis at all doses of CoQ tested (Figure 1C). We assayed the putative Aβ activation of neutral sphyngomielinase (nSMase), as previously described in neurons [30]. CoQ is also a potent antioxidant in plasma membrane [31] and we had previously demonstrated that higher CoQ levels in liver plasma membrane enhanced anti-oxidant protection by a mechanism involving the inhibition of nSMase [32]. However, Aβ did not activate nSMase in HUVECs (Figure S2).

**Figure 2:**
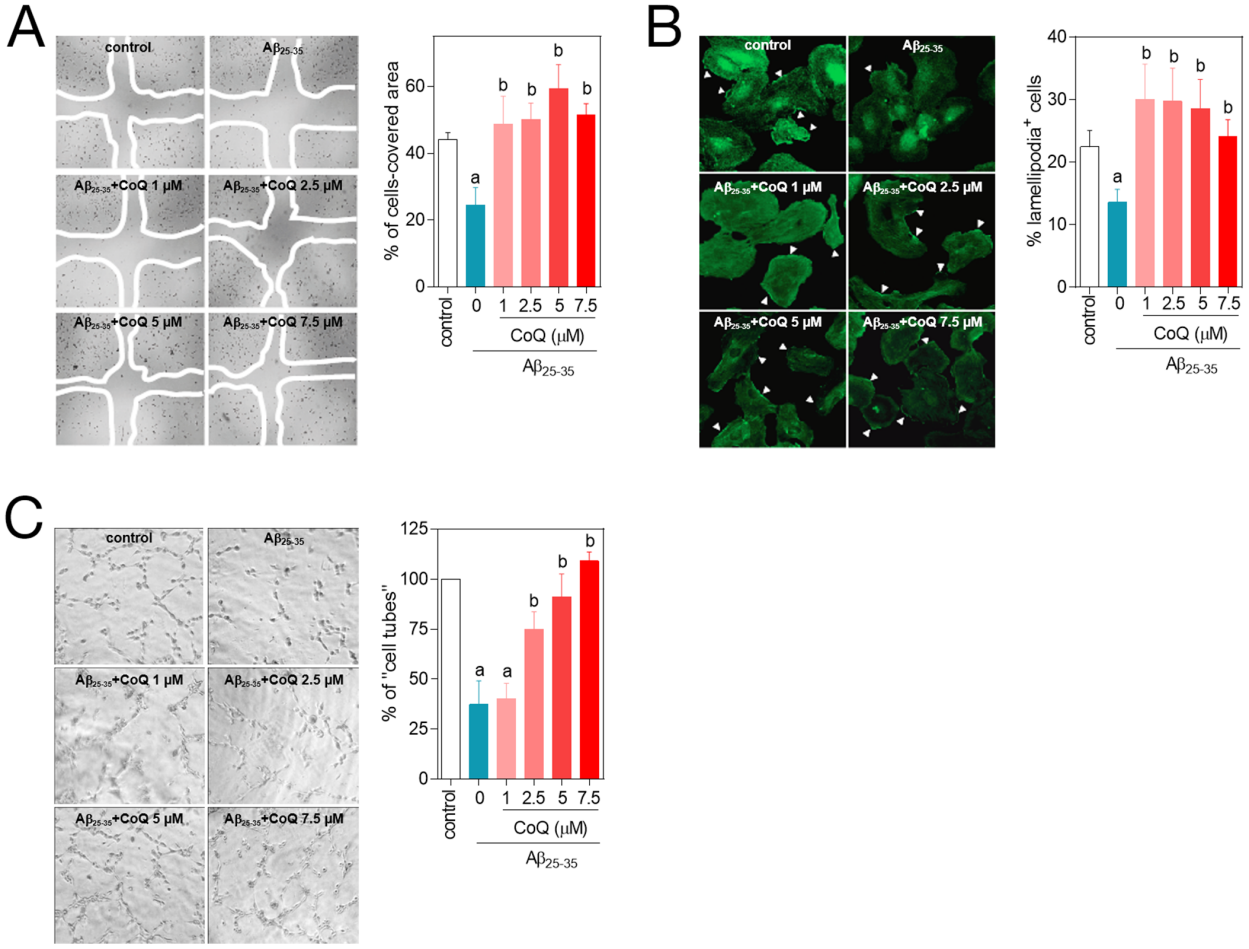
CoQ hinders β-amyloid inhibition of endothelial cells migration and tubes formation. HUVECs were incubated for 12 h with vehicle or increasing CoQ concentrations (1 to 7.5 µM) and treated for additional 24 h with 5 µM Aβ_25–35_. **A**) Cell migration was evaluated with the wound healing assay. Results show the percentage of wound recovered after 12 h for the indicated treatments (n = 3). **B**) Migrating cells were identified with fluorescence microscopy by immunostaining of lamellipodia structures with an anti-actin antibody. Results are expressed as percentage of lamellipodia^+^ cells (white arrows) cells *vs.* total (n = 3). **C**) Angiogenesis *in vitro* was determined by “cell tube” formation assays in Matrigel-coated wells. Results show the percentage of cell tubes *vs*. control after 6 h incubation with the indicated treatments (n = 3). a, *p*<0.05 *vs.* control; *b*, p<0.05 *vs.* Aβ_25–35_.

Aβ damages small blood vessels and impairs the blood supply in early Alzheimer's stages [4], while also increasing the permeability of the blood brain barrier [3] which reinforces the concept of an early endothelial degeneration in asymptomatic stages. There is also evidence suggesting that Aβ inhibits neovascularization *in vitro* [33], [34]. Thus, we tested the effect of CoQ on Aβ impaired angiogenesis *in vitro*, and also assaying effects on cell migration, a key step in vessel formation. Addition of Aβ resulted in a strong decrease in HUVECs migration, evaluated by a standard wound healing assay (Figure 2A), and similar results were obtained by quantifying lamellipodia positive (migrating) cells (Figure 2B). Pretreatment with CoQ completely abrogated the inhibitory effect of Aβ on HUVECs migration at all doses tested (Figures 2A,B). Similarly, Aβ produced a 60% reduction in the ability of HUVECs to form capillary-like cell tubes, and this effect was prevented by pretreatment with CoQ in a dose-dependent manner, (Figure 2C).

### CoQ prevents β-amyloid-dependent increase of O_2_^.−^, H_2_O_2_ and Ca^2+^ in endothelial cells

**Figure 3:**
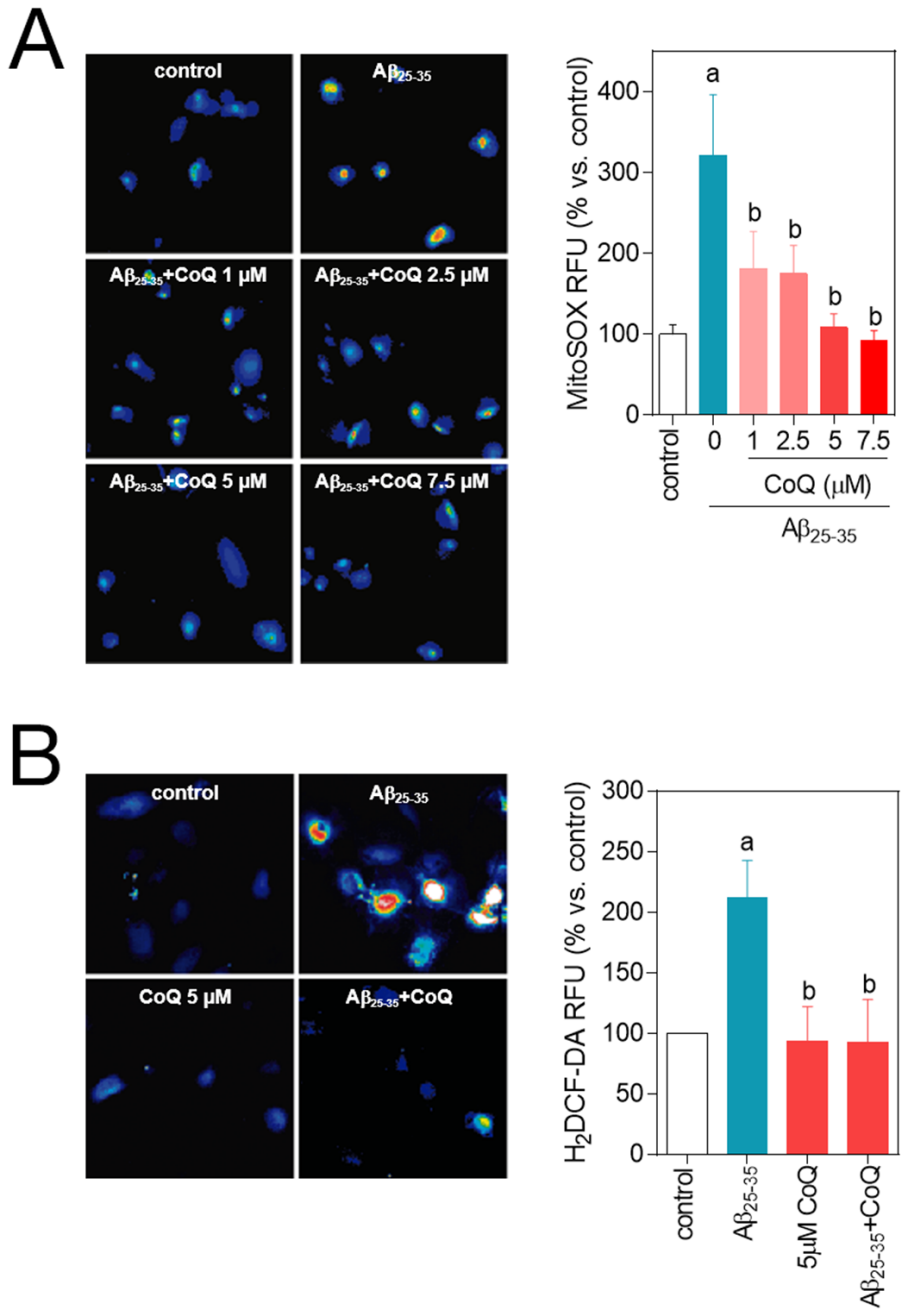
CoQ prevents β-amyloid-mediated increase in O_2_^.−^ and H_2_O_2_ levels in endothelial cells. HUVECs were incubated for 12 h with vehicle or increasing CoQ concentrations (1 to 7.5 µM) and treated for additional 24 h with 5 µM Aβ_25–35_. **A**) O_2_^.−^ levels were determined by fluorescence microscopy using the probe MitoSOX-AM. **B**) H_2_O_2_ level was determined by fluorescence microscopy with the probe H_2_DCF-DA. Results show the percentage of variation of fluorescence *vs*. control cells (n = 4). a, *p*<0.05 *vs.* control; *b*, p<0.05 *vs.* Aβ_25–35_.

**Figure 4:**
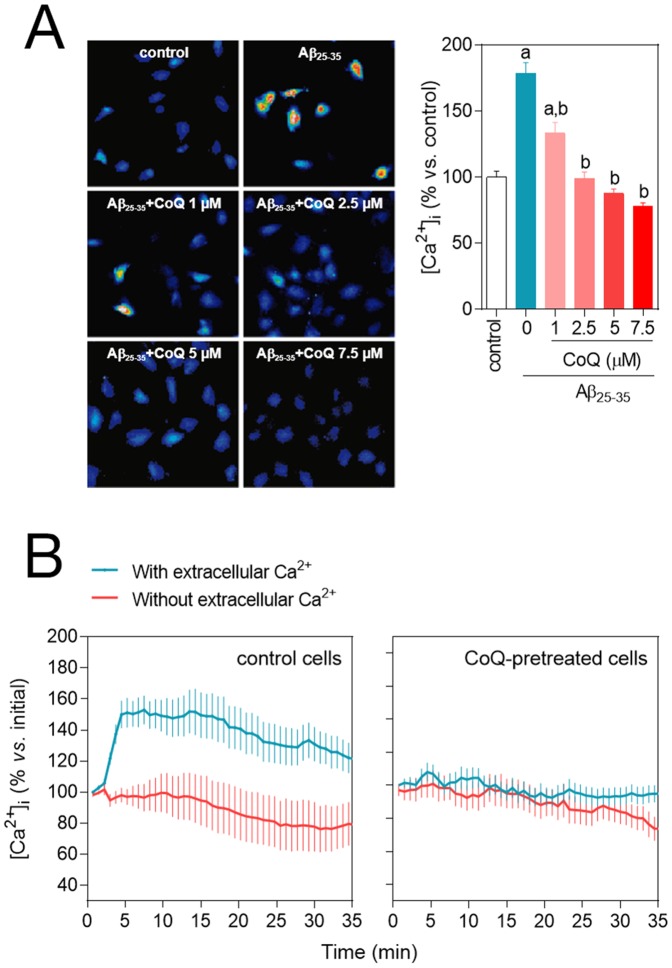
CoQ blocks β-amyloid-induced raise in the free cytosolic Ca^2+^ level in endothelial cells. HUVECs were incubated for 12 h with vehicle or CoQ (1 to 7.5 µM) and treated with 5 µM Aβ_25–35_. Ca^2+^ levels were determined by fluorescence microscopy with the probe Fluo-4-AM. **A**) Ca^2+^ was quantified after 3 h treatment with 5 µM Aβ_25–35_ in cells preincubated with CoQ. Results show the percentage of variation of fluorescence *vs*. control cells (n = 4). a, *p*<0.05 *vs.* control; *b*, p<0.05 *vs.* Aβ_25–35_. **B**) Changes in Ca^2+^ were monitored by time-lapse microscopy every 30 sec in cells pretreated with PBS (left graph) or 5 µM CoQ (right graph) in the presence (black line) or absence (grey line) of extracellular Ca^2+^. Aβ_25–35_ was added in min 1. Results show the averaged percentage of fluorescence variation *vs*. baseline before Aβ_25–35_ addition (n = 3).

The deleterious effect of Aβ in endothelial cells is due to an excess of O_2_^.−^ and H_2_O_2_ and altered calcium homeostasis [1], [5], [35], [36]. Thus, our results demonstrated that administration of 5 µM Aβ_25–35_ to HUVECs increased O_2_^.−^ (3-fold) and H_2_O_2_ (2-fold) levels *vs.* the untreated controls (Figure 3A,B). CoQ alone did not affect the basal levels of reactive oxygen species or free cytosolic Ca^2+^. However, preincubation with CoQ abated Aβ_25–35_–dependent increase of O_2_^.−^ at all doses tested, reaching control levels at 5–7.5 µM CoQ (Figure 3A). Similarly, Aβ failed to increase H_2_O_2_ levels in HUVECs preincubated with 5 µM CoQ (Figure 3B). In parallel, we tested the effect of CoQ pretreatment on Aβ-induced changes of Ca^2+^ homeostasis in HUVECs. Administration of 5 µM Aβ_25–35_ for 3 h produced a 75% increase of Ca^2+^ levels compared with basal conditions. Preincubation with CoQ reduced Aβ-dependent Ca^2+^ increase at all tested doses (Figure 4A). Simultaneous treatment with 5 µM Aβ_25–35_ and CoQ resulted in a similar Ca^2+^ increase than that induced by Aβ alone (Figure S1B), indicating that CoQ needs to be previously incorporated into the cell to impede Aβ action.

### CoQ prevents β-amyloid-induced entry of extracellular Ca^2+^ in endothelial cells

Though Aβ-mediated Ca^2+^ increase is well documented [5], [12], there is little study of the kinetics of this secondary messenger dynamics. To this end, we tested the effect of Aβ in control and CoQ pretreated cells using a real time approach. This technique revealed that the addition of 5 µM Aβ_25–35_ to control HUVECs induced a rapid Ca^2+^ increase from the extracellular media, reaching a maximum (150% above baseline) at 5 min from peptide administration (Figure 4B left, blue line). This Ca^2+^ increase was abolished by incubating the cells in Ca^2+^ free media (Figure 4B left, red line). Of note, extracellular Ca^2+^ entry induced by Aβ_25–35_ action was completely prevented in cells preincubated with 5 µM CoQ, as Ca^2+^ levels remained unaltered, with or without extracellular calcium (Figure 4B, right).

### β-amyloid cytotoxic effects are not prevented by the antioxidants tempol and α-tocopherol

**Figure 5:**
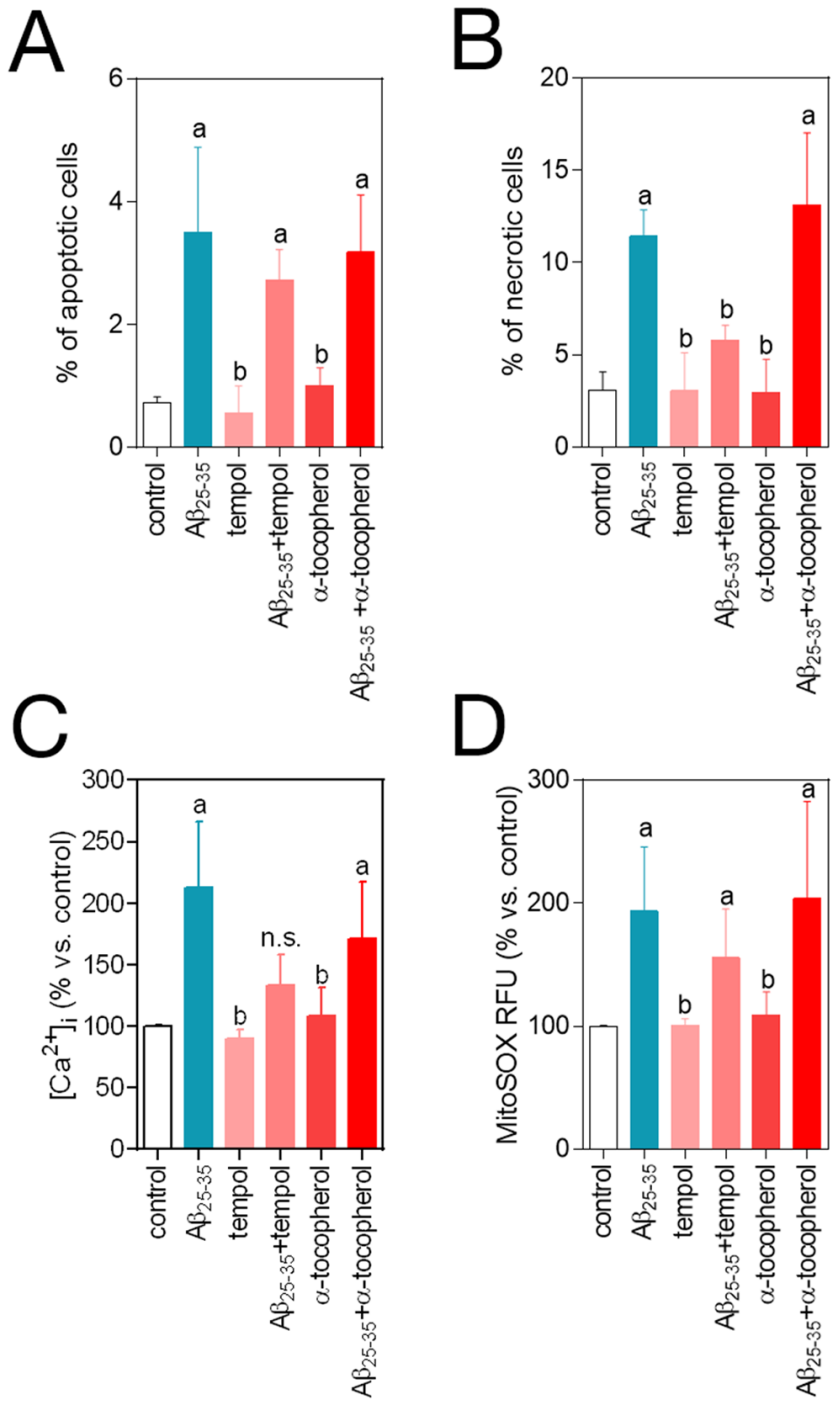
Effects of tempol and α-tocopherol on β-amyloid-induced apoptosis, necrosis, free cytosolic Ca^2+^ and O_2_^.−^. HUVECs were incubated for 12 h with vehicle, 1 mM tempol or 100 µM α-tocopherol, and then treated for additional 3–24 h with 5 µM Aβ_25–35_. **A**) Apoptosis was determined by DAPI staining and morphological analysis of nuclei. Results are expressed as the percentage of apoptotic *vs*. total nuclei (300 cells/treatment, n = 3). **B**) Necrosis was determined by staining with ethidium bromide and evaluated by qualitative fluorescence microscopy. Results are expressed as percentage of necrotic *vs.* total cells (n = 3). a, *p*<0.05 vs. control; b, p<0.05 *vs*. Aβ_25–35_. HUVECs were incubated for 12 h with vehicle, 1 mM tempol or 100 µM α-tocopherol and treated for additional 3 h with 5 µM Aβ_25–35_. **C**) Ca^2+^ levels were determined by fluorescence microscopy with the probe Fluo-4-AM. **D**) O_2_^.−^ levels were determined by fluorescence microscopy using the probe MitoSOX-AM. Results show the percentage of variation of fluorescence *vs*. control cells (n = 3). a, *p*<0.05 *vs.* control; *b*, p<0.05 *vs.* Aβ_25–35_.

In order to test if the protective effects of CoQ were imparted by its antioxidant properties, we studied the action of the antioxidants tempol and α-tocopherol on cell death, Ca^2+^ and O_2_^.−^ levels. Preincubation with 1 mM tempol or 100 µM α-tocopherol did not inhibit apoptosis triggered by 5 µM Aβ_25–35_ (Figure 5A), whereas only tempol was able to inhibit necrosis without reaching control levels (Figure 5B). Preincubation with any of the compounds was unable to restore Ca^2+^ and O_2_^.−^, obtaining only partial but not significant reductions in the case of tempol (Figure 5C, D).

### CoQ impedes NO decrease by β-amyloid in endothelial cells

**Figure 6:**
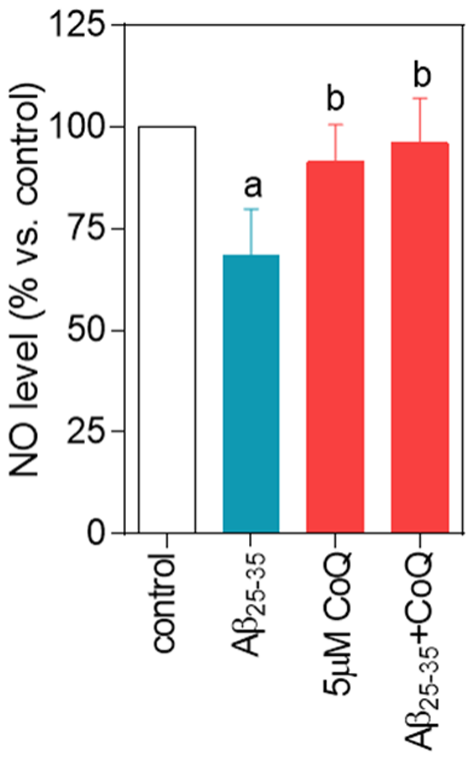
Nitric oxide decrease is prevented by CoQ treatment. HUVECs were incubated for 12 h with vehicle or 5 µM CoQ and treated for additional 24 h with 5 µM Aβ_25–35_. NO was determined in the supernatant by the Griess method. Results are expressed as percentage of NO *vs.* control (n = 4). a, *p*<0.05 vs. control; b, p<0.05 *vs*. Aβ_25–35_.

NO is dampened under oxidative stress conditions, due to its reaction with O_2_^.−^, resulting in the formation of peroxynitrite, and a subsequent increase in oxidative and nitrosative stress that alters endothelial cells function [35]. Our results, obtained with the Griess method, showed that a 24 h treatment of HUVECs with 5 µM Aβ_25–35_ decreased the production of NO by 40%. Pretreatment with 5 µM CoQ avoided this inhibitory effect of Aβ on NO production (Figure 6), which compares with the reduction in O_2_^.−^ and H_2_O_2_ by CoQ pretreatment, as shown above.

### CoQ prevents β-amyloid opening of the mPTP in endothelial cells

**Figure 7:**
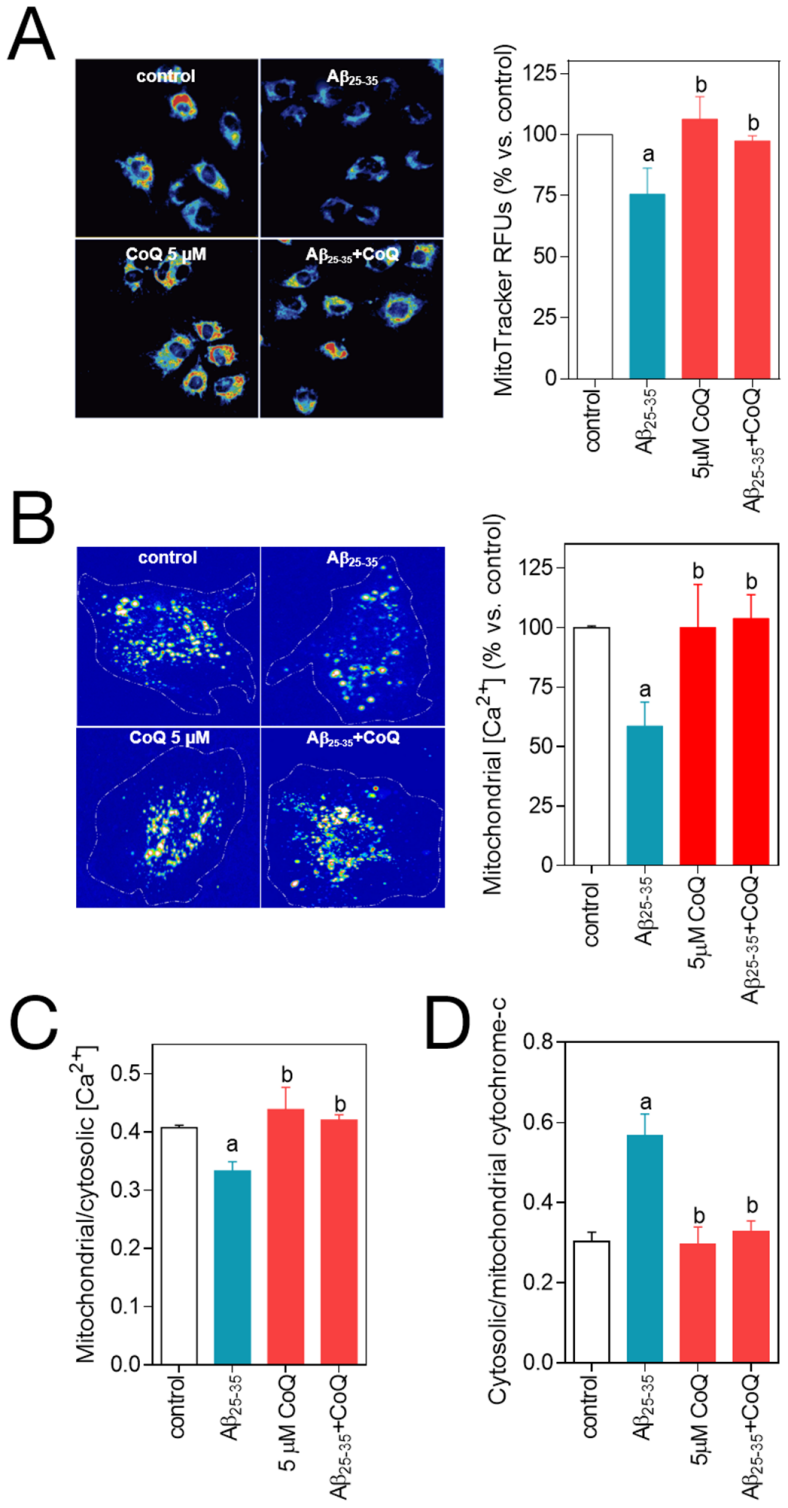
CoQ impedes β-amyloid-induced mPTP opening. HUVECs were incubated for 12 h with vehicle or 5 µM CoQ and treated for additional 3–24 h with 5 µM Aβ_25–35_. The effect of the treatments on mPTP opening was quantified indirectly by measuring MitoTracker Deep Red fluorescence, mitochondrial Ca^2+^ and cytochrome *c* release. After treatment with 5 µM Aβ_25–35_, mitochondria were loaded with MitoTracker Deep Red **A**), Calcein-AM and CoCl_2_ **B**) and MitoTracker Deep Red plus Fluo-4 **C**). Fluorescence intensity for each probe was determined by fluorescence microscope in living cells (n = 3/4). Mitochondrial Ca^2+^ levels were calculated by quenching the cytosolic Calcein-AM signal with CoCl_2_ **B**) and by colocalization of Fluo-4 and MitoTracker and furfher image processing with ImageJ **C**). Cells were loaded with Mitotracker Deep Red and immunostained with an anti-cytochrome *c* **D**). The amount of cytochrome *c* in cytosol was calculated by colocalization and image processing with ImageJ (n = 3). Results show the percentage of relative fluorescence units (RFUs) *vs.* control cells or the ratio between cytosolic/mitochondrial Fluo-4-AM or cytochrome *c* RFUs level. *a*, *p*<0.05 *vs.* control; *b*, p<0.05 *vs.* Aβ_25–35_.

Aβ-mediated rise in O_2_^.−^, H_2_O_2_ and Ca^2+^ is linked to the opening of the mitochondrial permeability transition pore, which releases mitochondrial content into the cytosol, including the cytochrome *c* from the inter-membrane space, and initiates cell death. As CoQ inhibits mPTP opening in neurons [16], [17], we speculated a similar mechanism could operate in endothelial cells injured by Aβ. We evaluated the mPTP by different strategies. We tested the effect of 5 µM Aβ_25–35_ on MitoTracker fluorescence and the level of mitochondrial Ca^2+^ in HUVECs. MitoTracker staining revealed a 25% reduction in the signal after 24 h treatment with 5 µM Aβ_25–35_ peptide (Figure 7A, Figure S3), which indicates a change in mitochondrial membrane potential, as MitoTracker intensity depends on mitochondrial membrane polarization. The 3 h treatment with 5 µM Aβ_25–35_ peptide was accompanied by a significant decrease in mitochondrial Ca^2+^ (Figure 7B,C, Figure S3), which supports an alteration in mitochondrial permeability. Both MitoTracker intensity signal and mitochondrial Ca^2+^ levels remained unaltered in HUVECs pretreated with 5 µM CoQ (Figure 7A,B,C, Figure S3). Furthermore, mPTP opening is reinforced by the release of cytochrome *c* to the cytosol. Addition of 5 µM Aβ_25–35_ to control HUVECs resulted into a strong release of cytochrome *c*, reaching a 2-fold increase in the cytosolic/mitochondrial ratio *vs.* untreated cells (Figure 7D; Figure S4). Cytochrome c release was completely abolished in HUVECs preloaded with 5 µM CoQ (Figure 7D, Figure S4).

### CoQ reduces β-amyloid entry and accumulation into endothelial cell mitochondria

**Figure 8:**
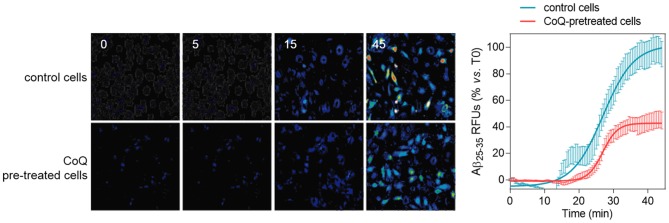
CoQ delays and decreases β-amyloid incorporation into endothelial cells. HUVECs were incubated for 12 h with vehicle or 5 µM CoQ. Changes in HiLyte Fluor-labeled Aβ_25–35_ were monitored by time-lapse microscopy every 30 sec in cells pretreated with PBS or 5 µM CoQ. Fluorescent peptide (5 µM) was added in min 1. Pictures show the variation of fluorescence at different time points, from 0 to 45 min. Graph shows fluorescence dynamics, expressed as normalized relative fluorescence units (RFUs), (n = 3).

**Figure 9:**
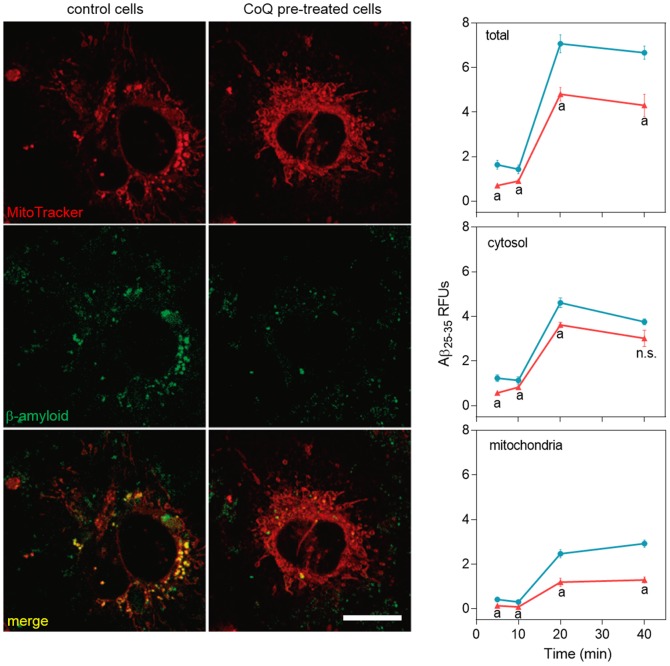
CoQ lessens β-amyloid incorporation into endothelial cells mitochondria. HUVECs were incubated for 12 h with vehicle or 5 µM CoQ, loaded with Mitotracker Deep Red, and then treated with 5 µM HiLyte Fluor-labeled Aβ_25–35_ for 0, 10, 20 and 40 min. Pictures were immediately acquired by confocal microscopy in living cells and show the staining with Mitotracker Deep Red, HiLyte Fluor-labeled Aβ_25–35_ (green) and merges, in control (left) and CoQ incubated cells (right) at 40 min from Aβ_25–35_ addition. Colocalization and image processing were performed with ImageJ. Graphs show HiLyte Fluor-labeled Aβ_25–35_ relative fluorescence units (RFUs) *vs.* time in the whole cell (total; upper graph), cyotosol (middle graph) and mitochondria (bottom graph) in control (black line) and CoQ incubated cells (grey line). n = 3; a, p<0.005 *vs.* Aβ_25–35_.

We next tested the effect of CoQ on the incorporation of fluorescent Aβ_25–35_ peptides into HUVECs and its trafficking and accumulation into mitochondria, using fluorescence and confocal microscopy in living cells. Our results showed that fluorescent Aβ_25–35_ signal surpassed the control cells threshold 15 m after peptide administration, reaching a plateau at 40 min (Figure 8, blue line). Preincubation of HUVECs with 5 µM CoQ resulted in a 10 m delay in the incorporation, accompanied by a 60% reduction in the maximum signal as compared with control cells (Figure 8, red line). These epifluorescence results were reproduced by confocal microscopy, which showed a 45% reduction in the level of fluorescent Aβ_25–35_ accumulation in whole CoQ pretreated HUVECs 40 m after peptide administration (Figure 9, upper graph). Co-staining with MitoTracker revealed that this difference was not due to an inhibition of Aβ entry into the cytosol (Figure 9, middle graph), but to its accumulation into the mitochondria (Figure 9, bottom graph). Nevertheless, cytosolic Aβ fluorescence was reduced in CoQ treated cells up to 20 m after Aβ addition, whereas differences to control cells lacked statistical significance after 40 min (Figure 9, middle graph). On the other hand, fluorescent Aβ was significantly decreased in the mitochondrial compartment at only 5 m after Aβ addition to CoQ pretreated cells, and this difference increased 3-fold at longer incubation times (Figure 9, bottom graph). Confocal microscopy images depicted in Figure 9 are representative cells incubated for 40 min with fluorescent Aβ. Taken together, these results demonstrate that CoQ delays and reduces the entry of Aβ into HUVECs and also significantly dampens trafficking and incorporation into the mitochondria.

### CoQ restores the metabolic profile altered by β-amyloid in endothelial cells

**Figure 10:**
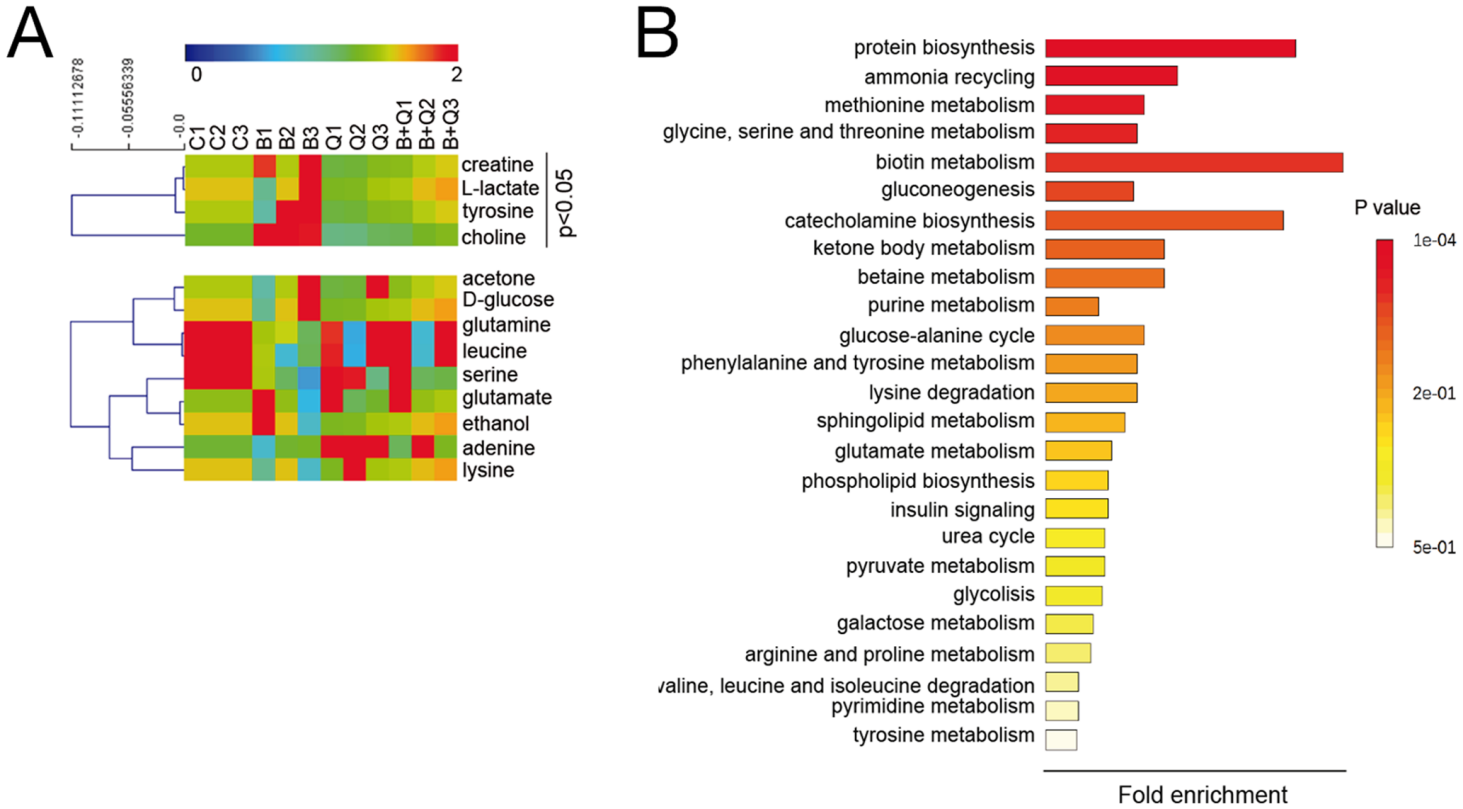
CoQ prevents metabolic changes induced by β-amyloid in endothelial cells. HUVECs were incubated for 12 h with vehicle or 5 µM CoQ and treated with 5 µM Aβ_25–35_. Samples were sonicated, lyophilized and dissolved in 40 µl D_2_O. ^1^H NMR spectra were registered with a Nanoprobe at 400 MHz resonance frequency. Metabolites were identified with Chenomx Profiler and by means of 2D homo- and heteronuclear NMR experiments. Differential analysis and classification was performed with MEV4 and pathways enrichment with MetaboAnalist. **A**) Heatmap and clustering of metabolites identified in HUVECs (n = 3). p<0.05 where indicated. **B**) Pathways enrichment based on the identified metabolites. Results show the averaged fold change vs. vehicle-treated cells (n = 3). a, *p*<0.05 *vs.* control; *b*, p<0.05 *vs.* Aβ_25–35_.

Recent studies using murine models of Alzheimer's disease revealed a profound alteration in brain metabolite profiles, which occurs prior to the deposition of Aβ plaques and the appearance of early behavioral changes [37]. Indeed, these changes reflect an increased mitochondrial stress and are related to alterations in Krebs cycle, energy transfer, carbohydrate, neurotransmitter, and amino acid metabolic pathways [37]. On this rationale and based on our previous results (see above), we questioned, first, if Aβ exerts a similar action in endothelial cells and, second, if CoQ could restore those possible Aβ-induced changes to control levels. Using a RMN-based metabolomic approach, we identified 13 metabolites (Figure 10A). Enrichment analysis revealed the alteration of several pathways including those related to protein biosynthesis, biotin metabolism and catecholamine biosynthesis, among others (Figure 10B). Indeed, addition of 5 µM Aβ_25–35_ to HUVECs for 24 h resulted in an increase in adenine, tyrosine, L-lactate, creatine and choline levels in 1.3, 1.4, 1.6, 1.4 and 1.75 fold of control, respectively (Figure 10A). Preincubation with 5 µM CoQ abolished the effect of Aβ, lowering metabolite levels to control levels, with the exception of adenine (Figure 10A).

## Discussion

Vascular degeneration is initiated by soluble Aβ in early, asymptomatic, Alzheimer's disease stages [2]. The deleterious effect of soluble Aβ in endothelial cells involves its incorporation into the membranes, uptake and trafficking to the mitochondria. This process results in an excess of superoxide free radicals and H_2_O_2_ plus the deregulation of calcium homeostasis, inducing cell death by apoptosis and necrosis. This damage impairs endothelial cell migration, thus creating gaps that compromise microvessel function [1], [35], [36]. In this study, we show that the lipophilic antioxidant CoQ emerges as an attractive candidate to prevent the deleterious effects of Aβ on endothelial cells in early Alzheimer's disease stages, which could significantly delay the progression of the pathology. Importantly, we found that CoQ effects are cytoprotective and should be administered before endothelial damage is irreversible.

CoQ cytoprotection involves the blockade of Aβ action at several steps, some of them shared by other antioxidant molecules, but others unique or not described to date. Indeed, previous investigations demonstrated that free radical scavengers prevented, at least in part, Aβ-induced toxicity [2]. Similarly, we have found that the antioxidants tempol and α-tocopherol at high doses only partially reversed Aβ-induced apoptosis and necrosis (Figure 5). On the other hand, CoQ cytoprotective effects are robust and maximal at 5 µM, a concentration which lies within a range reached *in vivo* after oral supplementation [38].

Our results show, for the first time, that CoQ reduces and delays Aβ incorporation and accumulation into the cell, which is paralleled by a reduction in the influx of extracellular Ca^2+^. It is known that soluble Aβ incorporates into the plasma membrane forming channels that produce ion leakiness, thus mediating Ca^2+^ entry into the cytosol [12]–[14], [39]. It is noteworthy that the protective effect of CoQ is not mediated by a simple chemical interaction with Aβ, because the simultaneous addition of CoQ and Aβ did not result in protection of HUVECs from Aβ toxicity. Rather, HUVECs should be preincubated with CoQ for this protective effect to be observed. Treatment of HUVEC with CoQ (1–10 µM) for 12 h increased its intracellular levels in a concentration-dependent manner [27]. Exogenous CoQ is initially accumulated in the endo-lysosomal compartment, and then rapidly becomes incorporated mainly to mitochondria-associated membranes and mitochondria, whereas its distribution among endomembranes and the plasma membrane is a late event, requiring 12 hours [40]. In addition, a functional intracellular vesicular trafficking is needed for CoQ to reach various cellular compartments [40]. This suggest that its inhibitory effect on Aβ uptake in endothelial cells does not involve the impairment of endocytic mechanisms. Moreover, the 25–35 fragment and the Aβ are highly hydrophobic peptides which are inserted into the membrane hydrocarbon core, and their incorporation depends on the lipid composition of the bilayer [39], [41]. CoQ is incorporated into all cell membranes [40] and disposes the long isoprenoid tail in the hydrophobic midplane of the lipid bilayer in such a way that phospholipid polar heads are in tight contact with the last isoprenoid unit of the CoQ hydrophobic tail [42]. Based on this, we hypothesize that CoQ could block, via steric hindrance, Aβ incorporation into the plasma membrane and Ca^2+^ entry, acting as a “cellular armor” and reducing the formation of channels.

The inhibition of the extracellular calcium influx by the addition of verapamil or pimozide (two calcium channels blockers) to endothelial cell culture medium only partially prevented the Aβ toxicity [5], indicating that Aβ also can release calcium from intracellular stores to increase the cytosolic calcium level. Mitochondria are major intracellular calcium stores and we demonstrated here that Aβ is quickly driven to mitochondria in HUVECs, deregulating calcium homeostasis. Preincubation of HUVECs with CoQ reduced Aβ uptake and mainly the Aβ accumulation into mitochondrion, which is paralleled by a blockade of the intracellular calcium influx induced by Aβ and the reestablishment of the mitochondrial calcium to control levels, which was otherwise significantly decreased under Aβ insult.

It has been demonstrated, both *in vitro* and *in vivo*, that Aβ is localized to the mitochondrial cristae of neurons [19] and that the sole mitochondria-specific Aβ accumulation is sufficient to cause ROS elevation and mitochondrial dysfunction, leading to mPTP opening and cytochrome *c* release, and causing cellular toxicity and death [16]–[18]. In this sense, a CoQ binding site regulates the aperture of the mPTP [28], and it has been reported that CoQ is effective at inhibiting mPTP opening induced by H_2_O_2_ in epithelial cells [29] or by Aβ in neurons [16], [17]. Our results show that CoQ protection from Aβ-induced deleterious effects in endothelial cells also involves these mechanisms. CoQ pretreatment reduced Aβ trafficking and accumulation into the mitochondria (Figure 9), impeding both mPTP opening and cytochrome *c* release, which was accompanied by a reduction in O_2_^.−^ and H_2_O_2_ levels. Furthermore, O_2_^.−^ and H_2_O_2_ reduction resulted in an increase of NO levels. As previously described in neurons, Aβ trafficking to the mitochondria increases ROS levels, which react with NO to produce peroxynitrite, inactivating MnSOD by nitration and generating additional free radicals that over-damage mitochondria [35], [43]. Furthermore, CoQ located in extra-mitochondrial membranes, such as in the Golgi compartment, may be an important cofactor to maintain eNOS in a coupled conformation required to produce physiological NO and, eventually, quench leaking-uncoupled electrons [44].

Metabolomics data reinforce the CoQ improvement of Aβ-impaired mitochondrial function. Indeed, Aβ treatment resulted in an increase of creatine levels (Figure 10), which, as reported previously, could be due to an inhibition of creatine kinase by oxidative stress-mediated carbonylation, thus disturbing mitochondrial bioenergetics [45]. Aβ also increased L-lactate level (Figure 10), which indicates a shift to fermentative metabolism, reinforcing Aβ-induced mitochondrial damage [37]. Our results show that pretreatment of endothelial cells with CoQ restored both creatine and L-lactate to control levels, indicating a normalization of mitochondrial function.

Aβ-increased ROS and Ca^2+^ levels in endothelial cells are translated into increased necrosis and apoptosis. Thus, we found an increased level of choline in Aβ-treated cells which, as indicated previously for retina pericytes, could be consequence of a high phosphatidylcholine hydrolysis due to the pro-oxidant effect of Aβ peptide that drives to necrotic cell death [46]. Pretreatment with CoQ restored choline to control levels, and also inhibited endothelial necrosis and apoptosis. In an *in vivo* setting, endothelial cell death in microvessels increases permeability and compromises oxygen delivery and nutrient exchange. Moreover, our results show that Aβ also interfered with the ability of endothelial cells to promote wound repair, as manifested by decreased motility and impaired tube-like structure formation. These processes are implicated in the disturbance of the endothelial integrity because, endothelial cells extruded lamellipodia to move and rapidly close the wound during repair processes [47]. Aβ_25–35_ addition to HUVECs resulted in a marked decrease in lamellipodia extrusion and a significant delay in wound closure, but preincubation with CoQ reversed the inhibition of the cellular motility. These findings suggest that CoQ can protect microvasculature against the Aβ_25–35_-induced endothelial cell death, but can also improve the restoration of the endothelial function by re-endothelization in pathological conditions associated to AD, reducing blood draining and improving oxygenation and nutrients delivery, thus, impacting on neuronal function.

Although AD is, by definition, a non-vascular dementia, vascular comorbidity may be present in the 30-60% of AD patients [20], making compounds that halt vascular oxidative stress putative candidates for therapeutical interventions. Augmented levels of ROS are common factors in all major vascular diseases [48]. Oral CoQ supplementation has been used recently in clinical trials to improve the endothelial function in type II diabetes and cardiovascular diseases [21], [22], [49], [50], and a meta-analysis of the randomized controlled trials concluded that CoQ supplementation is associated with significant improvement in endothelial function [22]. It has been proposed a plasma threshold of 2.5 µM, above which positive effects can be observed [51]. Reported plasma CoQ in healthy people ranged from 0.40 to 1.91 µM, but after daily oral supplementation with 200–300 mg of CoQ, plasma CoQ concentration increase up to 3–5 µM [38], the same CoQ concentration range used in our experiments. These data suggest that CoQ supplementation could be useful in therapy to protect endothelial cells *in vivo* against Aβ injury.

In conclusion, our results reported herein provide the first *in vitro* evidence that CoQ inhibits the uptake and mitochondrial trafficking of Aβ peptide in human endothelial cells, also alleviating Aβ-induced oxidative injury, cell death and decreased motility. CoQ emerges as an attractive molecule to prevent AD-associated endothelial dysfunction in high risk populations during early asymptomatic stages of the disease.

## Materials and Methods

### Reagents

Coenzyme Q10 was generously provided by Kaneka Corporation. Human Aβ_25–35_ and tempol were purchased from Tocris. Human Aβ_25–35_ labelled with HiLyte Fluor 488 was obtained from Anaspec. The fluorescent probes, Fluo-4 AM, H_2_DCF-DA, MitoSOX-AM, MitoTracker Deep Red, Calcein-AM and Hoescht were obtained from LifeSicences. Alpha tocopherol was acquired from Sigma-Aldrich.

### Cell culture

Human umbilical vein endothelial cells (HUVECs; Clonetics, Lonza) were maintained in complete EGM-2 medium (Clonetics, Lonza) containing 20% FBS and 1% antibiotic/antimycotic, at 37°C and 5% CO_2_. Experiments were performed in DMEM containing 20% FBS. All cells used in this study were up to the 15th passage.

### Determination of apoptosis, necrosis and viability

Cells were seeded in 96 well plates and incubated for 12 h with vehicle (controls), CoQ ranging from 1 to 7.5 µM, 1 µM tempol or 100 µM α-tocopherol. The medium was removed and Aβ_25–35_ was added to 5 µM. Where indicated, CoQ and Aβ_25–35_ where added simultaneously to the cultures. After 24 h, cells were incubated with 10 µg/ml EtBr and 1 µM Calcein-AM. Viable (green) and necrotic cells (red) were determined by fluorescence microscopy with a Nikon TiU microscope (Nikon, Tokyo, Japan) using a 20× objective. Immediately after image acquisition, cells were fixed and permeabilized for 2 min in ice-cold methanol and stained with 1 µg/ml Hoescht. Apoptotic nuclei were determined according morphological criteria. For necrosis, viability and apoptosis, results are expressed as percentage *vs.* total (n = 4). Additionally, apoptosis was determined by cell flow cytometry. To this end, cells were plated onto 6-well plates and treated as described above. Then, cells were labeled with an anti-annexin V antibody (Bender MedSystems) and apoptotic cells were quantified with a cell flow cytometer FACSCalibur (BD Biosciences) following the manufacturer's instructions. Results are shown as percentage of apoptotic cells *vs.* total (n = 4).

### Assays for migration and cell tube formation

HUVECs were plated in 12-well plates, cultured to confluence and then serum starved for 12 h in medium containing vehicle (control) or 5 µM CoQ. A cross-scratch was done in the monolayer with a 10 µl pipet tip and the medium was replaced by a fresh medium containing 5 µM Aβ_25–35_. Percentage of wound closure was calculated by measuring on each image, with ImageJ, the open area free of cells immediately after doing the scratch and at 24 h. Results shown are average of n = 3.

To analyze cytoskeleton reorganization, cells were treated as indicated above and then fixed for 5 min in cold methanol, incubated for 30 min in blocking buffer, stained with the monoclonal antibody for β-actin (Sigma) and detected with an AlexaFluor488-conjugated anti-mouse antibody (Life Sciences). Images were obtained using a Nikon TiU microscope (20× objective). To quantify the effects of the treatments on actin reorganization and thus, on cell mobility, cells with a static and a migratory (lamellipodia positive) phenotype were counted in a double blind procedure. At least 200 cells in 4 independent experiments were counted for each group, and cells with migratory phenotype were considered positive. Results are expressed as percentage of positive cells normalized *vs.* control.

For *in vitro* angiogenesis, HUVECs incubated for 12 h with vehicle (control) or CoQ in 24 well plates were detached and plated onto GFR Matrigel-precoated 96-well plates, adding 5 µM Aβ_25–35_ to the culture medium. Cells were incubated for 6 h and then, the number of cell-tubes was counted from phase-contrast microscopy images. Results were calculated as percentage of cell-tubes *vs*. control (with neither CoQ nor Aβ, n = 4).

### Determination of O_2_^.−^, H_2_O_2_ and Ca^2+^ in single cells

Mitochondrial O_2_^.−^, total H_2_O_2_ and free cytosolic Ca^2+^ levels were determined with the fluorescent probes MitoSox, H_2_DCF-DA and Fluo-4 (Life Sciences), respectively. Cells were seeded in 96 well plates and incubated for 12 h with vehicle (controls) or CoQ ranging from 1–7.5 µM, 1 mM tempol or 100 µM α-tocopherol. Medium was removed and cells were incubated for 3 h in fresh medium containing 5 µM Aβ_25–35_ peptide. Cells were then loaded for 30 m with 1 µM of the fluorescent probe (one independent probe per assay), washed in fresh medium and imaged in a Nikon TiU microscope (20× objective). Images were analyzed and processed with ImageJ. Results show the percentage of cell signal *vs.* control (n = 4).

The effect of CoQ on Aβ_25–35_-induced Ca^2+^ level was determined using a real time epifluorescence approach. Cells were grown onto 35 mm plates and treated for 12 h with vehicle (control) or 5 µM CoQ. Then, cells were loaded for 30 min with 1 µM Fluo-4 and media was replaced by Hank's balanced solution with/without Ca^2+^, to assess whether Aβ_25–35_ effect was due to Ca^2+^ entry or release from intracellular stores. Cells where imaged in a Nikon TiU microscope (20x objective). The baseline was established within the first minute of recording and after that, 5 µM Aβ_25–35_ was added to the plate. Epifluorescence images were recorded every 15 s up to 35 m. Series were analyzed with ImageJ. Results show the percent of averaged profiles *vs*. baseline, for each treatment (n = 3).

### NO determination

NO level was assessed by the quantification of nitrite accumulation in the culture medium. Cells were seeded in 12 well plates and incubated for 12 h with vehicle (control) or 5 µM CoQ. Total nitrite accumulated was measured using the modified Griess reagent, according the indications of the manufacturer (Sigma-Aldrich). Results were evaluated by spectrophotometry (BioRad iMark) at 540 nm and expressed as percentage *vs.* control (n = 4).

### Mitochondrial Ca^2+^ and cytochrome *c* levels

Mitochondrial Ca^2+^ was determined by staining the cells with Calcein-AM and quenching the cytosolic Calcein signal with CoCl_2_. To this end, cells were seeded in 8 well μ-slides (Ibidi) and incubated for 12 h with vehicle (control) or 5 µM CoQ. The medium was removed and cells were incubated for 3 h in fresh medium containing 5 µM Aβ_25–35_ peptide. Then, cells were loaded for 30 min with 1 µM Calcein-AM and 1 mM CoCl_2_, washed in fresh medium and imaged in a Nikon TiU microscope (60x objective). Results, calculated with ImageJ, are expressed as the percentage of mitochondrial Calcein signal *vs.* control (n = 3). Alternatively, mitochondrial Ca^2+^ was evaluated by co-staining cells with Fluo-4 and the fluorescent mitochondrial probe Mitotracker DeepRed (Life Sciences), which signal is sensitive to mitochondrial potential changes. To this end, cells were seeded in 96 well plates and incubated for 12 h with vehicle (control) or 5 µM CoQ. The medium was removed and cells were incubated for 3 h in fresh medium containing 5 µM Aβ_25–35_ peptide. Then, cells were loaded for 30 min with Fluo-4 and Mitotracker DeepRed (1 µM each), washed in fresh medium and imaged in a Nikon TiU microscope (20× objective). Mitotracker DeepRed signal was directly quantified with ImageJ. Results show the percentage of cell signal *vs.* control (n = 4). The amount of mitochondrial Ca^2+^ was calculated by image analysis, subtracting the Fluo-4 signal colocalizing with Mitotracker to the whole cell Fluo-4 signal. This processing was done with ImageJ. Results are expressed as the ratio between mitochondrial and total Ca^2+^ for each treatment (n = 4).

Cytochrome *c* subcellular localization was assessed by labeling mitochondria with Mitotracker DeepRed and immunolabeling cytochrome *c* with a specific monoclonal antibody (BD Biosciences). Briefly, cells seeded in 96 well plates were incubated for 12 h with vehicle (control) or 5 µM CoQ. Medium was removed and cells were incubated for 24 h in fresh medium containing 5 µM Aβ_25–35_ peptide. Then, cells were loaded for 30 min with 1 µM Mitotracker DeepRed, fixed for 2 min in cold methanol, and stained for 30 min with fluorescein labeled anti-cytochrome *c* antibody (1∶200). Cells were washed and immediately imaged in a Nikon TiU microscope (20× objective). The amount of mitochondrial cytochrome *c* was calculated by image analysis, subtracting the whole cell cytochrome *c* signal to the cytochrome *c* signal colocalizing with Mitotracker. Image processing was performed with ImageJ. Results are expressed as the ratio between cytosolic and mitochondrial cytochrome *c* level for each treatment (n = 4).

### sMASE activity

Cells were seeded in 12 well plates and incubated for 24 h with vehicle (controls) or 5 µM CoQ and then incubated for 24 h in fresh medium containing 5 µM Aβ_25–35_ peptide. Cells were detached and nSMAse activity was determined from cell extracts as described in earlier studies [32].

### Aβ_25–35_ incorporation into HUVECs

The effect of CoQ on Aβ_25–35_ incorporation into HUVECs was determined by both fluorescence and confocal approaches. In a first approach, cells were grown onto 35 mm plates and treated for 24 with vehicle (controls) or 5 µM CoQ. Then, plates were mounted onto the stage of a Nikon TiU microscope under a 20x objective. A concentration of 5 µM Aβ_25–35_ peptide labeled with HiLyte Fluor 488 was added and fluorescence was recorded every 30 sec up to 45 min. Time-series were analyzed with ImageJ. Results show averaged cell-fluorescence profiles *vs*. baseline (n = 3) and data fit to exponential growth curves. For confocal studies, cells were grown onto gelatin-coated glass-bottom 35 mm plates and treated for 24 with vehicle (controls) or 5 µM CoQ. Then, cells were stained with 1 µM Mitotracker DeepRed and incubated with 5 µM Aβ_25–35_ labeled with HiLyte Fluor 488 for 5, 10, 20 and 40 min. After incubation, cells were washed and plates mounted onto the stage of a Zeiss LSM5 confocal microscope under a 40x objective. Images were sequentially acquired for green (Aβ_25–35_ peptide) and red (Mitotracker DeepRed) channels and processed with ImageJ to calculate the signal of fluorescent Aβ_25–35_ in the whole cell, cytosol and mitochondria. Results show the averaged cell Aβ_25–35_ fluorescence (n = 3).

### Metabolomics

Cells were grown onto 25 cm^2^ flasks and treated for 12 with vehicle (control) or 5 µM CoQ and then incubated for 24 h in fresh medium containing 5 µM Aβ_25–35_ peptide. Cells were detached, rinsed twice in PBS and homogenized by sonication in deuterated D_2_O (99.98% atom % D) (Sigma-Aldrich). Extracts were lyophilized and resuspended again in 40 µl of deuterated water (99.994 atom % D) (Sigma-Aldrich). NMR spectra were performed on a Varian VNMRS-400 NMR system using a nanoprobe with rotors of 40 µl. 1D and 2D NMR spectra were recorded for peak identification. The NMR spectra were processed with Mestrenova (Mestrelab Research) and peaks were also identified with Chenomx Profiler (Chenomx Inc.). Clustering and paired analysis for changes in the metabolites profile were assessed with the free software MEV 4.9 (http://www.tm4.org/mev.html). Pathways enrichment was analyzed using the platform MetaboAnalist 2.0 (http://www.metaboanalyst.ca/MetaboAnalyst/faces/Home.jsp).

### Statistical analysis

Data are expressed as mean ± S.E.M. obtained from, at least, three separate, independent experiments carried out on different days with different cell preparations. Statistical analysis was carried out with Graphpad Prism 6, using Student's t-test followed by a Mann–Whitney statistical test or one-way ANOVA (Kruskal–Wallis test) followed by a statistical test for multiple comparisons (Dunn's test). Differences were considered significant at *p*<0.05.

## Supporting Information

Figure S1 **Co-administration of CoQ affects neither free cytosolic Ca^2+^ nor apoptosis increase by β-amyloid.** **A**) HUVECs were co-incubated for 12 h with CoQ and Aβ_25–35_ (5 µM each). Apoptosis was determined by DAPI staining and morphological analysis of nuclei. Results are expressed as the percentage of apoptotic *vs*. total nuclei (300 cells/treatment, n = 3). **B**) HUVECs were co-incubated for 3 h with vehicle and CoQ and Aβ_25–35_ peptide (5 µM each). Ca^2+^ levels were determined by fluorescence microscopy with the probe Fluo-4-AM. Results show the percentage of variation of fluorescence *vs*. control cells (n = 3). a, *p*<0.05 *vs.* control.
(TIF)

Figure S2 **Effect of β-amyloid peptide and CoQ on n-SMase activity.** HUVECs were incubated for 12 h with 5 µM CoQ, treated for additional 24 h with 5 µM Aβ_25–35_ peptide and then washed and pelleted. Neutral SMase activity was assayed as described in [32]. (n = 4).
(TIF)

Figure S3 **Representative pictures of mitochondrial Ca^2+^ quantification.** HUVECs were incubated for 12 h with vehicle or 5 µM CoQ and treated for additional 3 h with 5 µM Aβ_25–35_. Ca^2+^ levels were measured with Fluo-4-AM. Mitochondria were labeled with MitoTracker Deep Red. Images were acquired with an inverted fluorescence microscope and processed with ImageJ. For each picture, a mask corresponding to mitochondria was subtracted from total Ca^2+^ image to obtain a value of total-mitochondrial Ca^2+^.
(TIF)

Figure S4 **Representative pictures of cytosolic cytochrome** ***c*** **quantification.** HUVECs were incubated for 12 h with 5 µM CoQ and treated for additional 24 h with 5 µM Aβ_25–35_. Cytochrome *c* was determined by ICC (green). Mitochondria were labeled with MitoTracker Deep Red. Images were acquired with an inverted fluorescence microscope and processed with ImageJ. For each picture, a mask corresponding to mitochondria was subtracted to total cytochrome *c* image, to obtain a value of the cytosolic fraction.
(TIF)
