# Supplementary figures and images for: Coenzyme Q10 Protects Human Endothelial Cells from β-Amyloid Uptake and Oxidative Stress-Induced Injury
Source: PLoS One. 2014 Oct 1;9(10):e109223. doi: 10.1371/journal.pone.0109223 (PMC4182835; doi:10.1371/journal.pone.0109223)

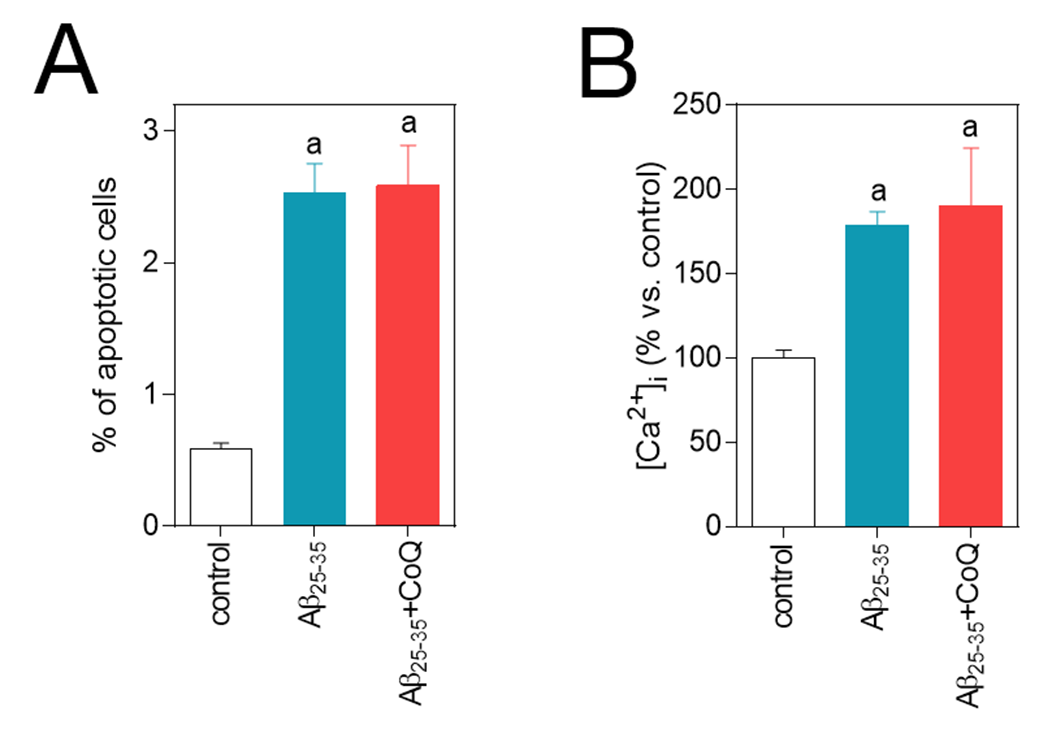

Supplement: Figure S1 — Co-administration of CoQ affects neither free cytosolic Ca2+ nor apoptosis increase by β-amyloid. A) HUVECs were co-incubated for 12 h with CoQ and Aβ25–35 (5 µM each). Apoptosis was determined by DAPI staining and morphological analysis of nuclei. Results are expressed as the percentage of apoptotic vs. total nuclei (300 cells/treatment, n = 3). B) HUVECs were co-incubated for 3 h with vehicle and CoQ and Aβ25–35 peptide (5 µM each). Ca2+ levels were determined by fluorescence microscopy with the probe Fluo-4-AM. Results show the percentage of variation of fluorescence vs. control cells (n = 3). a, p<0.05 vs. control. (TIF) [file pone.0109223.s001.tif]

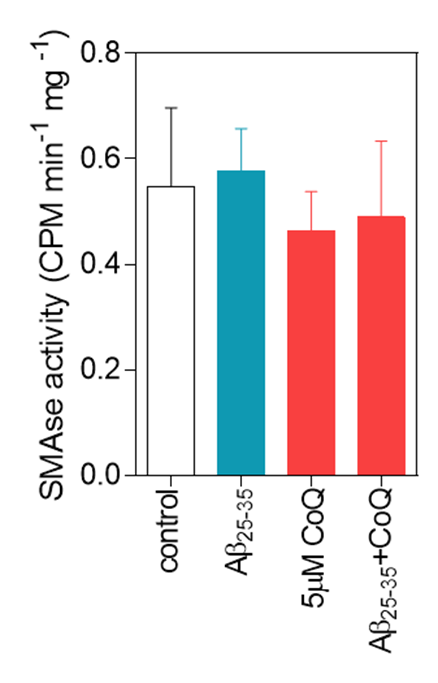

Supplement: Figure S2 — Effect of β-amyloid peptide and CoQ on n-SMase activity. HUVECs were incubated for 12 h with 5 µM CoQ, treated for additional 24 h with 5 µM Aβ25–35 peptide and then washed and pelleted. Neutral SMase activity was assayed as described in [32]. (n = 4). (TIF) [file pone.0109223.s002.tif]

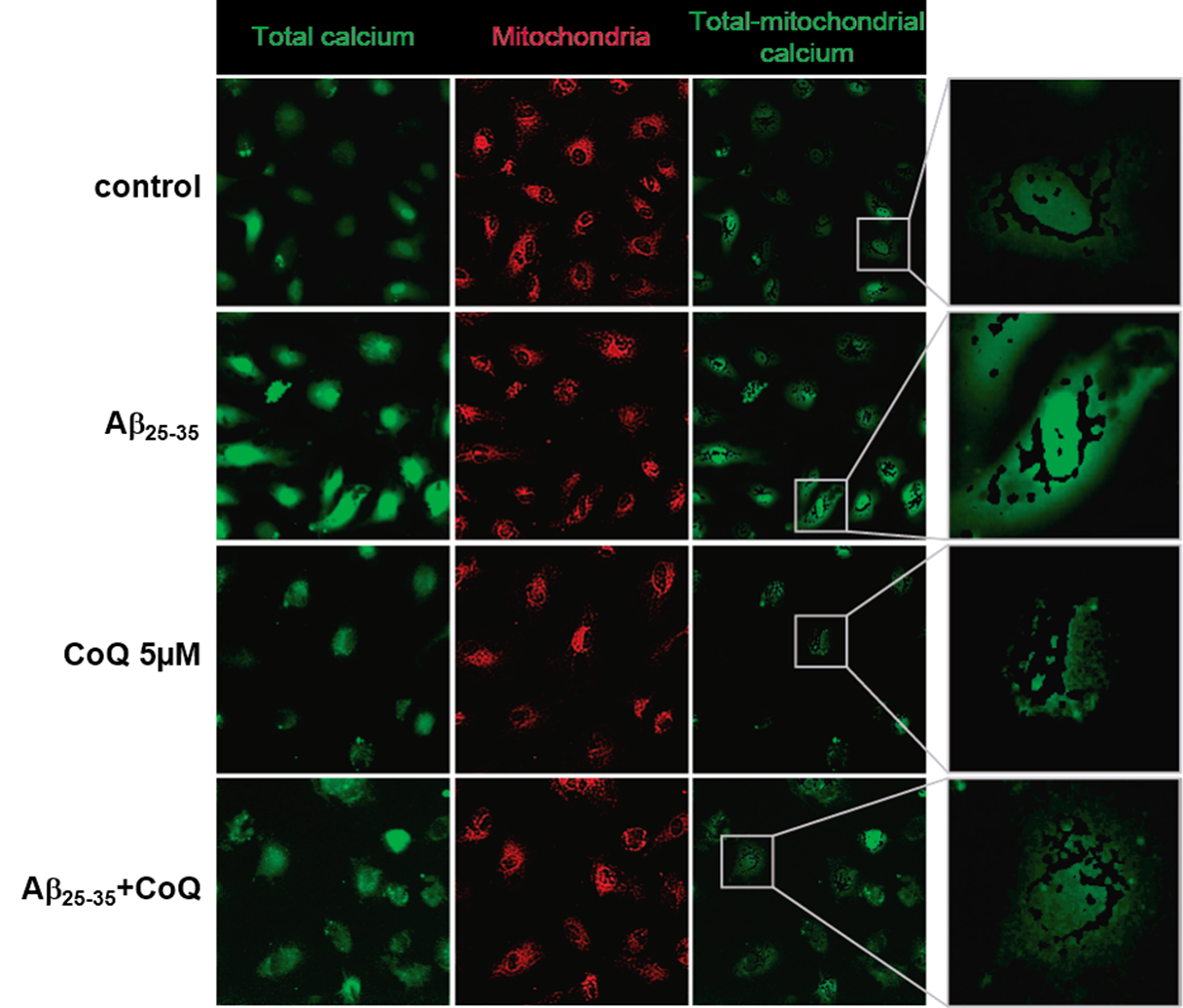

Supplement: Figure S3 — Representative pictures of mitochondrial Ca2+ quantification. HUVECs were incubated for 12 h with vehicle or 5 µM CoQ and treated for additional 3 h with 5 µM Aβ25–35. Ca2+ levels were measured with Fluo-4-AM. Mitochondria were labeled with MitoTracker Deep Red. Images were acquired with an inverted fluorescence microscope and processed with ImageJ. For each picture, a mask corresponding to mitochondria was subtracted from total Ca2+ image to obtain a value of total-mitochondrial Ca2+. (TIF) [file pone.0109223.s003.tif]

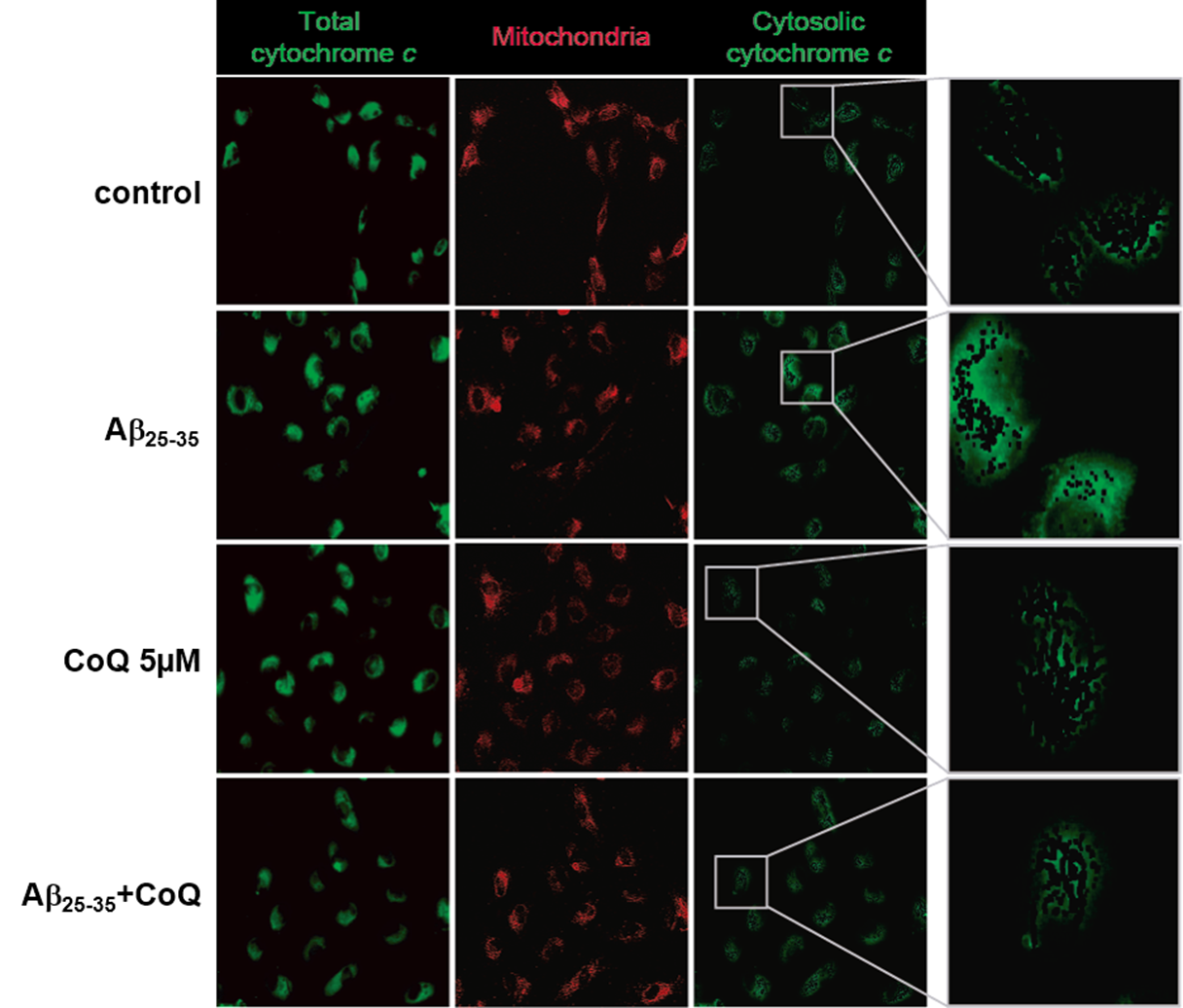

Supplement: Figure S4 — Representative pictures of cytosolic cytochrome c quantification. HUVECs were incubated for 12 h with 5 µM CoQ and treated for additional 24 h with 5 µM Aβ25–35. Cytochrome c was determined by ICC (green). Mitochondria were labeled with MitoTracker Deep Red. Images were acquired with an inverted fluorescence microscope and processed with ImageJ. For each picture, a mask corresponding to mitochondria was subtracted to total cytochrome c image, to obtain a value of the cytosolic fraction. (TIF) [file pone.0109223.s004.tif]
